# Draft genome sequence of *Thermovorax subterraneus* 70B^T^, a thermophile isolated from a geothermally active underground mine that produces hydrogen

**DOI:** 10.1016/j.dib.2022.108695

**Published:** 2022-10-25

**Authors:** Kian Mau Goh, Kok Jun Liew, Saleha Shahar, Iffah Izzati Zakaria, Ummirul Mukminin Kahar

**Affiliations:** a Department of Biosciences, Faculty of Science, Universiti Teknologi Malaysia, 81310 Skudai, Johor, Malaysia; b Malaysia Genome and Vaccine Institute, National Institutes of Biotechnology Malaysia, Jalan Bangi, 43000 Kajang, Selangor, Malaysia

**Keywords:** Clostridia, Geothermal, Hydrogen-producing bacterium, Underground mine, Thermophile, Thermosediminibacteraceae, Thermovorax, Whole genome sequencing

## Abstract

*Thermovorax subterraneus* 70B^T^ is a thermophile found in a geothermically active underground mine. The strain 70B^T^ belongs to the class of *Clostridia*, order of *Thermosediminibacterales*, and family of *Thermosediminibacteraceae*. Strain 70B^T^ was the only type strain since the genus was discovered >10 years ago. Strain 70B^T^ was compared to strains from other genera in terms of its phenotypics, chemotaxonomics, and phylogenetics (16S rRNA gene) in previous studies. However, the genome sequence of this strain has not been described. We herein described the genome sequence of strain 70B^T^. In total, the assembled genome of strain 70B^T^ has a size of 2,451,552 bp, contributed by 44 contigs, with a coverage of 445X, a N50 of 86,294 bp, and a GC% of 43.8. A total of 2,540 genes were encoded in the genome, including 2,431 protein-coding sequences, 46 pseudogenes, and 63 RNA genes. Through the Cluster of Orthologous Groups (COGs) analysis, a total of 2,404 protein-coding genes were functionally assigned to COGs in the genome of strain 70B^T^. Among the members of *Thermosediminibacteraceae* family, strain 70B^T^ has the closest relationship to *Caldanaerovirga acetigignens* JW/SA-NV4^T^ based on the genome-to-genome comparison indexes (i.e., ANI, dDDH, AAI, and POCP). An earlier study reported that strain 70B^T^ could produce hydrogen. We discovered genes encoding [FeFe] hydrogenase through gene mining analysis. For future research, this genome data will be used as a reference for all matters pertaining to the genus *Thermovorax* and family *Thermosediminibacteraceae*.

**Specifications Table**

**Table:** 

Subject	Microbiology
Specific subject area	Microbial Genomics
Type of data	Genome sequence data Table Figure
How the data were acquired	Whole genome sequencing using Illumina NovaSeq 6000 system
Data format	Raw, filtered, assembled, and analyzed.
Description of data collection	High quality genome of *Thermovorax subterraneus* 70B^T^ was purchased from the Leibniz Institute DSMZ-German Collection of Microorganisms and Cell Cultures GmbH (Braunschweig, Germany). A paired-end library was prepared using the NEBNext Ultra II DNA library preparation kit for Illumina (New England BioLabs, Ipswich, MA, USA), according to the manufacturer's protocol. Whole genome sequencing was performed using the Illumina NovaSeq 6000 system with 150-bp paired-end reads. Raw reads were filtered using Trimmomatic v0.39. *De novo* assembly was performed using SOAPdenovo v2.40, SPAdes v3.15.3, and ABySS v2.3.4. The genome annotation was conducted using the NCBI Prokaryotic Genome Annotation Pipeline (PGAP) v5.30.
Data source location	Institution: Universiti Teknologi Malaysia City/Town/Region: Skudai, Johor Country: Malaysia Bacteria isolation source: A geothermally active underground mine, Japan
Data accessibility	The data is hosted on a public repository. NCBI BioProjectAccession Number: PRJNA797675 NCBI BioSample Accession Number: SAMN25026613 NCBI GenBank Accession Number: JAKIHM000000000 NCBI Sequence Read Archive (SRA) Accession Number: SRX13800199
Related research article	A.E. Mäkinen, A.H. Kaksonen, J.A. Puhakka, *Thermovorax subterraneus*, gen. nov., sp. nov., a thermophilic hydrogen-producing bacterium isolated from geothermally active underground mine, *Extremophiles*. 13 (2009) 505–510. https://doi.org/10.1007/s00792-009-0235-5

## Value of the Data

• The first draft genome sequence of *Thermovorax subterraneus* 70B^T^ can provide insight into the genetic diversity of the genus, species, and family *Thermosediminibacteraceae*.
• These genome data provide beneficial information for scientists looking to further explore the genus *Thermovorax*, as well as for species delineation if any other closely related strains are discovered in the future.
• Genome sequences of *Thermovorax subterraneus* 70B^T^ can be used to discover enzymes and gene clusters involved in hydrogen production.

## 1 Data Description

*Thermovorax subterraneus* 70B^T^ (= DSM 21563^T^ = JCM 15541^T^) was isolated from a geothermally active underground mine located in Japan [1]. Strain 70B^T^ is a Gram-positive, rod-shaped, and motile thermophile that grows optimally at 71°C with pH 7.0–7.5. A heat treatment analysis at 95°C for 25 minutes does not destroy the strain, indicating that it is heat stable. An earlier study had examined strain 70B^T^ phenotypically, chemotaxonomically, and phylogenetically (16S rRNA gene) against close strains from other genera [1]. As of now, strain 70B^T^ is classified under the class *Clostridia*, the order *Thermosediminibacterales*, and the family *Thermosediminibacteraceae*. Since its discovery >10 years ago, strain 70B^T^ remains the only type strain in the genus. The aims of this sequencing project are to fill in any missing data regarding the type strain genome and to spark scientific interest in an underexplored genus.

**Fig. 1:**
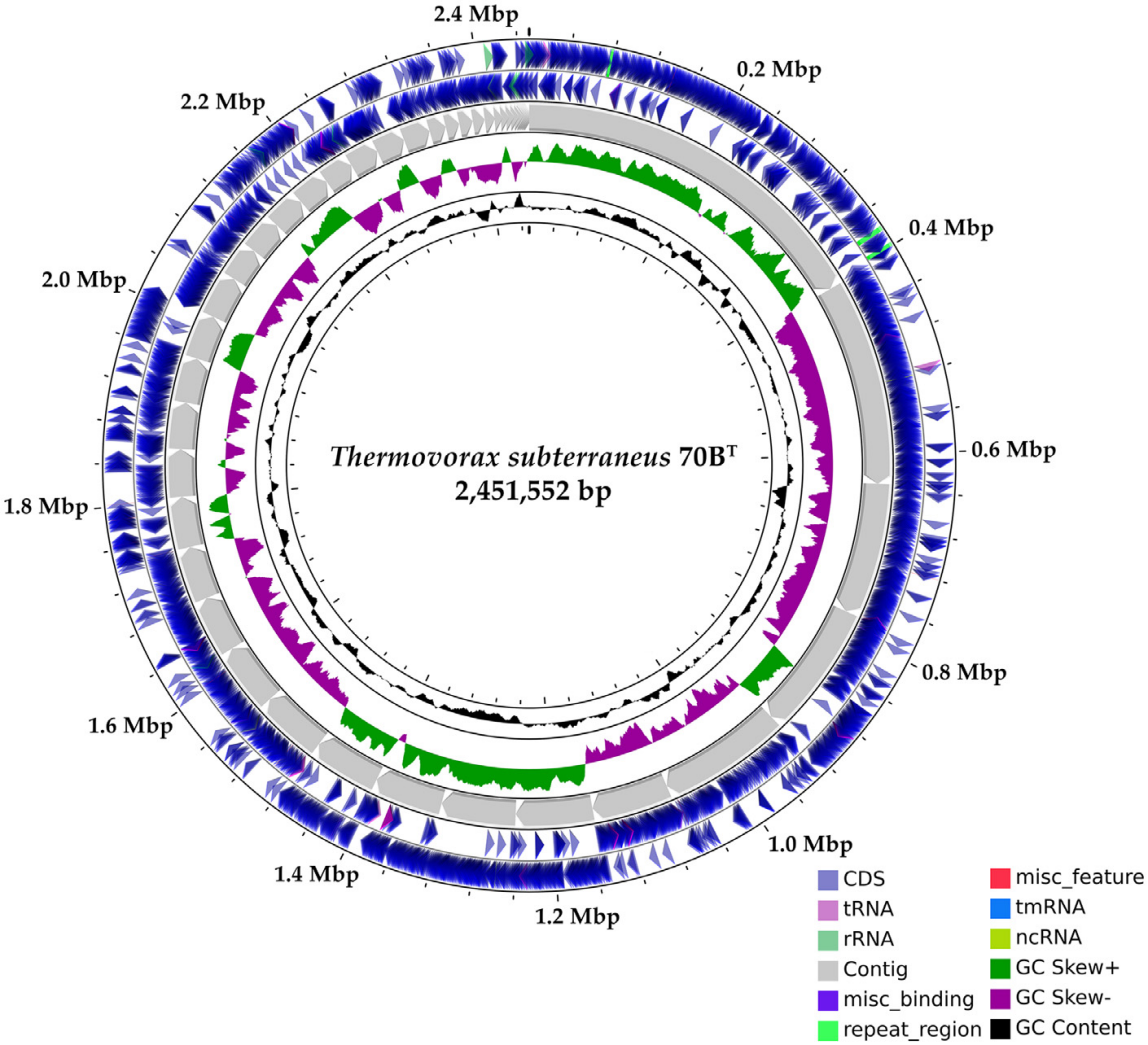
Circular genome map of *Thermovorax subterraneus* 70B^T^. From innermost to outermost circle: GC content, GC Skew of both forward and reverse strands, total number and size of each contigs, Open Reading Frames (ORFs) of both forward and reverse strands marked with respective genome features (i.e., CDS, tRNAs, rRNAs, and etc.).

The sequencer generated a total of 1.2 Gb in 4.0 million paired-ends reads. Following adapter trimming and low-quality read filtering, the sequence data was assembled into 44 contigs, with a size of 2,451,552 bp, a coverage of 445X, a N50 of 86,294 bp, and an average GC% of 43.8. Based on the NCBI Prokaryotic Genome Annotation Pipeline (PGAP) v5.30 annotation, the genome of strain 70B^T^ contains 2,540 genes, with 2,431 protein-coding sequences, 46 pseudogenes, and 63 RNA genes (50 tRNAs, four 5S rRNA, four 16S rRNA, one 23S rRNA, and four noncoding RNA genes). The genome map of strain 70B^T^ is shown in Fig. 1, and Table 1 compares its genome characteristics to those of other genomes in *Thermosediminibacteraceae* family.

**Table 1:** Genome characteristics of *Thermovorax subterraneus* 70B^T^ and other reported genome in the family *Thermosediminibacteraceae.*

Feature	TS	CA	FF	TL	TO	TG
Status	Draft	Draft	Draft	Draft	Complete	Draft
Coverage (X)	445	481	50	509	30	50
No. of contigs	44	38	107	72	1	47
Total length (bp)	2,451,552	2,264,710	2,439,065	2,344,789	2,280,035	2,282,800
N50 (bp)	86,294	145,277	61,803	71,562	2,280,035	142,665
GC%	43.8	43.3	45.8	47.8	46.8	38.0
Total genes	2,540	2,385	2,580	2,465	2,332	2,333
Protein-coding sequences	2,431	2,293	2,453	2,364	2,204	2,234
Pseudogenes	46	29	48	47	64	26
tRNAs	50	50	59	45	51	56
ncRNAs	4	4	4	4	4	4
rRNAs (5S, 16S, 23S)	9 (4, 4, 1)	9 (4, 2, 3)	16 (4, 6, 6)	5 (2, 2, 1)	9 (3, 3, 3)	13 (3, 6, 4)
Reference	This study	[2]	[3]	[4]	[4,5]	[6]

TS: *Thermovorax subterraneus* 70B^T^; CA: *Caldanaerovirga acetigignens* JW/SA-NV4^T^; FF: *Fervidicola ferrireducens* Y170; TL: *Thermosediminibacter litoriperuensis* JW/YJL-1230-7/2^T^; TO: *Thermosediminibacter oceani* JW/IW-1228P^T^; TG: *Thermovenabulum gondwanense* R270.

The protein-coding sequences of strain 70B^T^ were classified into different Cluster of Orthologous Groups (COGs) categories. Table 2 also includes the COGs distribution of those members from family *Thermosediminibacteraceae*. Strain 70B^T^ and other members have the highest COGs in the [S]–Function unknown category. This is then followed by [C]–Energy production and conversion, [E]–Amino acid transport and metabolism, [J]–Translation, ribosomal structure, and biogenesis, [K]–Transcription, and [L]–Replication, recombination, and repair. Each of these groups has more than 150 protein-coding sequences.

**Table 2:** Cluster of Orthologous Groups of protein (COGs) of *Thermovorax subterraneus* 70B^T^ and other members of family *Thermosediminibacteraceae*.

COGs Categories		TS	CA	FF	TL	TO	TG
**Cellular Processes and Signalling**							
[D]	Cell cycle control, cell division, chromosome partitioning	46 (1.91%)	46	46	49	49	45
[M]	Cell wall/membrane/envelope biogenesis	105 (4.37%)	105	122	113	113	96
[N]	Cell motility	78 (3.24%)	80	79	80	70	69
[O]	Post-translational modification, protein turnover, and chaperones	55 (2.29%)	54	58	65	52	58
[T]	Signal transduction mechanisms	99 (4.12%)	97	103	100	77	97
[U]	Intracellular trafficking, secretion, and vesicular transport	64 (2.66%)	59	70	68	57	51
[V]	Defence mechanisms	23 (0.96%)	0	30	24	27	20
[W]	Extracellular structures	1 (0.04%)	1	0	0	0	1
[Y]	Nuclear structure	0	0	0	0	0	0
[Z]	Cytoskeleton	1 (0.04%)	0	0	0	0	0
**Information Storage and Processing**							
[A]	RNA processing and modification	0	0	0	0	0	0
[B]	Chromatin structure and dynamics	1 (0.04%)	1	2	1	1	0
[J]	Translation, ribosomal structure, and biogenesis	170 (7.07%)	164	167	168	167	164
[K]	Transcription	162 (6.74%)	155	154	174	154	146
[L]	Replication, recombination, and repair	178 (7.40%)	152	243	151	149	127
**Metabolism**							
[C]	Energy production and conversion	218 (9.07%)	205	217	205	198	222
[E]	Amino acid transport and metabolism	174 (7.34%)	171	154	156	127	194
[F]	Nucleotide transport and metabolism	91 (3.79%)	88	93	86	76	94
[G]	Carbohydrate transport and metabolism	136 (5.66%)	127	105	135	108	103
[H]	Coenzyme transport and metabolism	151 (6.28%)	146	129	104	113	132
[I]	Lipid transport and metabolism	54 (2.25%)	40	43	37	43	55
[P]	Inorganic ion transport and metabolism	106 (4.41%)	123	109	130	126	103
[Q]	Secondary metabolites biosynthesis, transport, and catabolism	33 (1.37%)	33	25	28	26	27
**Poorly Characterized**							
[R]	General function prediction only	0	0	0	0	0	0
[S]	Function unknown	458 (19.05%)	424	431	435	383	429
Total		2,404 (100%)	2,271	2,380	2,309	2,116	2,233
Reference		This study	[2]	[3]	[4]	[4,5]	[6]

TS: *Thermovorax subterraneus* 70B^T^; CA: *Caldanaerovirga acetigignens* JW/SA-NV4^T^; FF: *Fervidicola ferrireducens* Y170; TL: *Thermosediminibacter litoriperuensis* JW/YJL-1230-7/2^T^; TO: *Thermosediminibacter oceani* JW/IW-1228P^T^; TG: *Thermovenabulum gondwanense* R270.

**Fig. 2:**
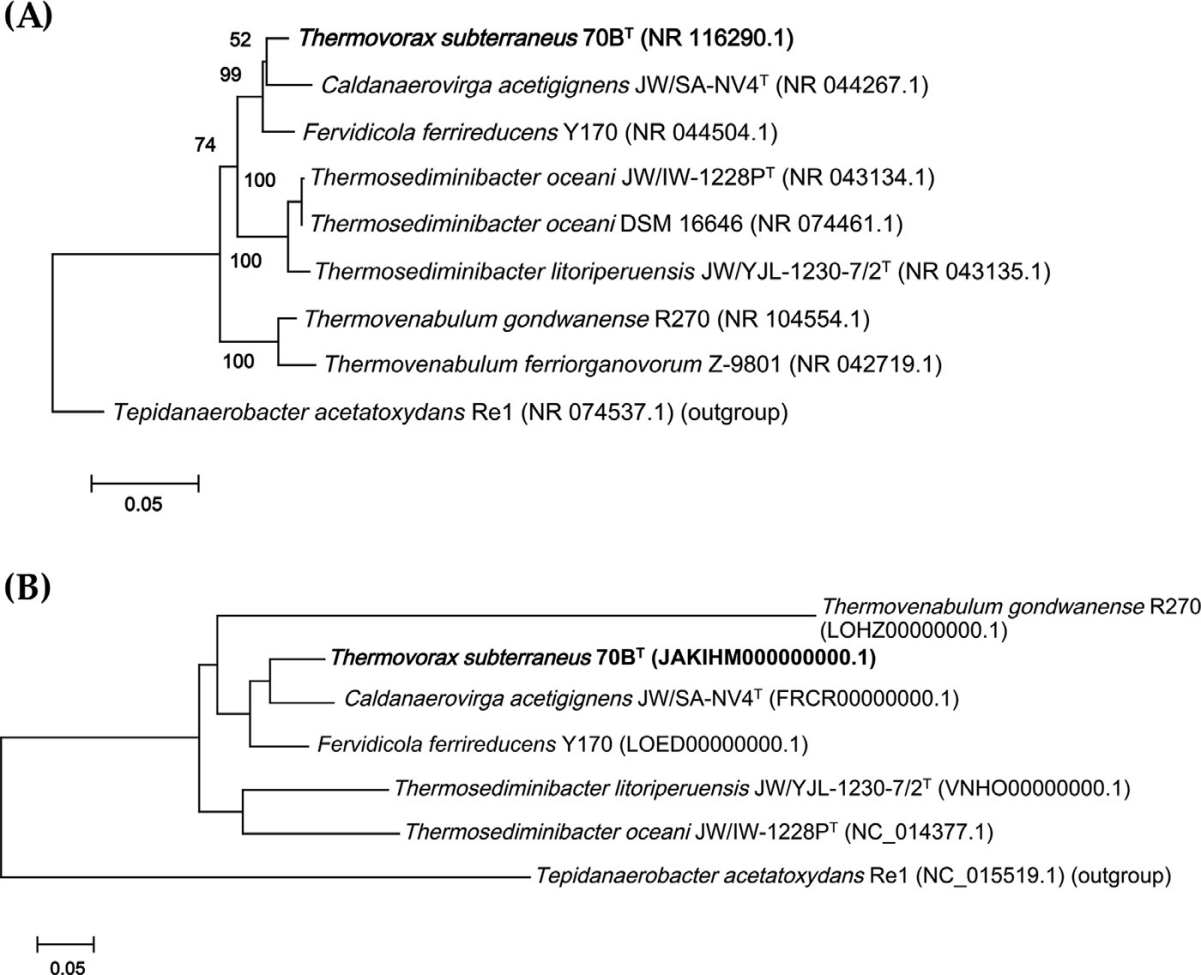
**(A)** Phylogenetic tree based on 16S rRNA gene sequences showing the relationship *Thermovorax subterraneus* 70B^T^ and representatives of family *Thermosediminibacteraceae.* The tree was constructed using a neighbour-joining method with 1,000 bootstrap replicates. **(B)** Phylogenomic trees of *Thermovorax subterraneus* 70B^T^ with other closer strains constructed using PhyloPhlAn.

In Table 3, we compared strain 70B^T^ to other strains of the family *Thermosediminibacteraceae* with available genomes. The average nucleotide identity index (ANI) values range from 68.9–92.6% and the digital DNA-DNA hybridization index (dDDH) values are between 18.6–48.6%. The homology index of amino acid sequences between strain 70B^T^ and other closest bacteria is 67.7–93.9% for average amino acid sequence identity (AAI) and 71.3–86.2% for percentage of conserved proteins (POCP). A phylogenetic tree of *Thermosediminibacteraceae* family based on 16S rRNA genes and a phylogenomic tree of all the available genomes are shown in Fig. 2A and B, respectively. According to our analysis, strain 70B^T^ is most closely related to *Caldanaerovirga acetigignens* JW/SA-NV4^T^ [2].

**Table 3:** Comparative genome analyses of *Thermovorax subterraneus* 70B^T^ against other species of *Thermosediminibacteraceae* family via 16S rRNA gene similarity, ANI, dDDH, AAI, and POCP.

Feature	TS	CA	FF	TL	TO	TG
16S rRNA gene similarity (%)	100	97.02	97.21	95.18	94.43	93.60
^a^ ANI (%)	100	92.60	89.72	73.93	73.94	68.96
^b^ dDDH (%)	100	48.60	39.20	18.60	19.40	21.60
^c^ AAI (%)	100	93.80	91.70	77.25	76.87	67.74
^d^ POCP (%)	100	86.23	81.14	74.24	71.25	72.08
Reference	This study	[2]	[3]	[4]	[4,5]	[6]

TS: *Thermovorax subterraneus* 70B^T^; CA: *Caldanaerovirga acetigignens* JW/SA-NV4^T^; FF: *Fervidicola ferrireducens* Y170; TL: *Thermosediminibacter litoriperuensis* JW/YJL-1230-7/2^T^; TO: *Thermosediminibacter oceani* JW/IW-1228P^T^; TG: *Thermovenabulum gondwanense* R270.

According to the research group that isolated strain 70B^T^ earlier, the bacterium was able to produce hydrogen gas [1]. Compared to mesophilic bacteria, thermophilic bacteria generate a higher yield of biohydrogen in dark fermentation [7]. Strain 70B^T^ fermented glucose primarily into H_2_, CO_2_, acetate, ethanol, and lactate [1]; however, it is unknown whether this strain able to utilize other substrates (i.e., starch waste). The hydrogenase enzymes in bacteria catalyze the reduction of protons into hydrogen during the anaerobic decomposition of organic compounds. It was determined that gene MCF6095860.1 encodes a transcriptional factor (TF) for hydrogenase system regulator. The sequence of this protein shares 46% sequence identity with the sequence of TM1266 TF from *Thermotoga maritima* with protein crystal structure deposited in PDB database (PDB ID: 2NZC). Besides, a group A-type [FeFe] hydrogenase (MCF6096026.1 and MCF6097398.1) is encoded in the genome. These hydrogenases contain a di-iron center and the active site is named the H-cluster. Several genes necessary for the formation of H-clusters are present, including several copies of the 4Fe-4S and 2Fe-2S binding proteins, carbon monoxide dehydrogenases (MCF6097400.1 and MCF6097401.1), as well as HydE, HydG, and the GTPase HydF (MCF6095948.1, MCF6096188.1 and MCF6096065.1). The gene encoding [NiFe] hydrogenase is not present in this bacterium.

## 2 Experimental Design, Materials and Methods

*Thermovorax subterraneus* 70B^T^ genomic DNA was purchased from the Leibniz Institute DSMZ-German Collection of Microorganisms and Cell Cultures GmbH (Braunschweig, Germany). A paired-end library was prepared using the NEBNext Ultra II DNA library preparation kit for Illumina (New England BioLabs, Ipswich, MA, USA), according to the manufacturer's protocol. DNA sequencing was performed using the NovaSeq 6000 system (Illumina, San Diego, CA, USA) with 150-bp paired-end reads. The sequence adaptors and low-quality reads were filtered using Trimmomatic v0.39 [8]. The clean reads were subjected to *de novo* assembly using SOAPdenovo v.2.40 [9], SPAdes v3.15.3 [10], and ABySS v2.3.4 [11]. Then, the assemblies were integrated using Contig Integrator for Sequence Assembly (CISA) v1.3 [12]. The gene prediction and annotation was conducted using NCBI Prokaryotic Genome Annotation Pipeline (PGAP) v5.30 [13]. Circular genome map was visualized using CGView server [14]. The protein-coding genes were clustered into functional groups using evolutionary genealogy of genes: Non-supervised Orthologous Groups (eggNOG)-mapper v2.1.7 [15]. Genome comparison between strain 70B^T^ and representatives strains with available genomes from family *Thermosediminibacteraceae* was carried out via (i) the average nucleotide identity (ANI) using Orthologous Average Nucleotide Identity Tool (OrthoANI) v0.93.1 [16], (ii) the digital DNA-DNA Hybridization (dDDH) calculated using Genome-to-Genome Distance Calculator v3.0 [17], (iii) the average amino acid sequence identity (AAI) analyzed using EzAAI v1.1 [18], and (iv) the Percentage Of Conserved Protein (POCP) calculated in a terminal shell using a deposited Ruby script in github (https://github.com/hoelzer/pocp) [19]. The genome-wide phylogenomics tree was constructed using PhyloPhlAn v3.0.2 [20]. Default parameters were used for all software tools unless stated otherwise.

## Ethics Statements

This work did not involve human subjects, animal experiments, and data collected from social media platforms.

## CRediT authorship contribution statement

**Kian Mau Goh:** Validation, Writing – review & editing, Funding acquisition, Project administration. **Kok Jun Liew:** Methodology, Data curation, Writing – original draft. **Saleha Shahar:** Funding acquisition, Writing – review & editing. **Iffah Izzati Zakaria:** Writing – review & editing, Funding acquisition. **Ummirul Mukminin Kahar:** Writing – review & editing, Funding acquisition.

## Declaration of Competing Interest

The authors declare that they have no known competing financial interests or personal relationships that could have appeared to influence the work reported in this paper.

## Data Availability

Thermovorax subterraneus strain DSM 21563, whole genome shotgun sequencing project (Original data) (NCBI).
